# Research on the Correlation between Serum Vitamin D Level and Cognitive Function in Parkinson's Disease in Plateau Areas

**DOI:** 10.1002/alz70856_099643

**Published:** 2025-12-24

**Authors:** Jialing Chen, Aiqin Zhu

**Affiliations:** ^1^ Institute of Geriatric, Qinghai Provincial Hospital, Qinghai University, Xining, Qinghai, China; ^2^ Graduate School of Qinghai University, Xining, China; ^3^ Qinghai Provincial Hospital, Qinghai University, Xining, China; ^4^ Clinical Research Center for Geriatric Diseases of Qinghai, Xining, China

## Abstract

**Background:**

To explore the impact of serum vitamin D level on the cognitive function of Parkinson's disease (PD) patients in hypoxia environment.

**Method:**

From September 2021 to September 2024, 231 PD patients (aged 66.88±11.10 years) were selected from the Parkinson's clinic of Qinghai Provincial People's Hospital(Average altitude:2300 meters). According to the Mini‐Mental State Examination (MMSE), they were divided into 66 the normal cognitive group (NC),114 mild cognitive impairment group (MCI), and 51 Parkinson's dementia group (PDD), Demographic data were collected, PD and cognition‐related scales were evaluated, and blood index were measured.

**Result:**

There were statistically significant differences in age, gender, education, family situation, occupation, smoking and hyperhidrosis among the three groups (*p* <0.05). Compared with the NC group, there were increased the total Unified Parkinson's Disease Rating Scale (UPDRS) score, UPDRS‐I, II, III, Hoehn‐Yahr (H‐Y) stage, Hamilton Depression Rating Scale (HAMD), Hamilton Anxiety Rating Scale (HAMA), Pittsburgh Sleep Quality Index (PSQI) and Activity of Daily Living (ADL) in MCI group and PDD group, while MMSE, Montreal Cognitive Assessment (MOCA), red blood cells, hemoglobin, folic acid, and vitamin B12 decreased (*p* <0.05). Parkinson's cognitive decline(PDCI) was positively correlated with age (r=0.033), hyperhidrosis (r=0.054), total UPDRS score (r=0.002) and H‐Y stage (r=0.502), while, negatively correlated with folic acid (r=‐0.189), vitamin D (r=‐0.039), red blood cells (r=‐0.188), and hemoglobin (r=‐0.004). Ordered regression display that UPDRS‐II (activities of daily living) (OR=1.100, 95%CI=1.021‐1.206) was a risk factor for PDCI, Vitamin D (OR=0.169, 95%CI=‐0.349‐0.271) was a protective factor (*p* <0.05).

**Conclusion:**

High serum vitamin D levels have a protective effect on PDCI, while a decline in ADL can lead to cognitive impairment in hypoxia environment.

